# Neuroprotective Effects and Mechanisms of Zhenlong Xingnao Capsule in *In Vivo* and *In Vitro* Models of Hypoxia

**DOI:** 10.3389/fphar.2019.01096

**Published:** 2019-09-26

**Authors:** Xia Wei, Qingfen Zhu, Na Liu, Lihua Xu, Sheng Wei, Zhiyun Fan, Changhua Sun, Yan Zhao, Mingqi Qiao, Jibiao Wu, Defu Hu, Yang Wang, Peng Sun

**Affiliations:** ^1^Department of Pharmacology and Toxicology, Shandong Institute for Food and Drug Control, Ji’nan, China; ^2^Key Laboratory of Traditional Chinese Medicine for Classical Theory, Ministry of Education, Shandong University of Traditional Chinese Medicine, Ji’nan, China; ^3^Laboratory of Ethnopharmacology, Institute of Integrated Traditional Chinese and Western Medicine, Xiangya Hospital, Central South University, Changsha, China

**Keywords:** Zhenlong Xingnao Capsule, middle cerebral artery occlusion model, cerebral ischemia-reperfusion injury, reactive oxygen species, BV-2 cell

## Abstract

Zhenlong Xingnao Capsule (ZXC) is a Tibetan medicine used to treat ischemic stroke. In this study, we determined the *in vitro* and *in vivo* effects of ZXC on reactive oxygen species (ROS) in a mouse BV-2 microglial cell hypoxia-reoxygenation and rat middle cerebral artery occlusion infarction models. We aimed to clarify the role of ZXC in cerebral ischemia protection; reveal amino acid neurotransmitter changes in the frontal cortex after drug intervention; determine mRNA and protein expression changes in Bcl-2, Bax, caspase-3, P38, and nuclear factor (NF)-кB in the frontal cortex and changes in antioxidant indices in the brain; and elucidate the mechanisms underlying ZXC action. After hypoxia-reoxygenation, ROS levels were significantly increased in BV-2 cells, and their levels decreased after treatment with ZXC. ZXC had protective effects on ischemic/anoxic injury *in vitro* and *in vivo* by downregulating the expressions of caspase-3 and NF-кB mRNA during ischemia and reperfusion and that of p38 and caspase-3 during acute ischemia and reperfusion as well as the steady-state levels of excitatory amino acids/inhibitory amino acids and by improving the total antioxidant capacity and total superoxide dismutase activities during ischemia. These findings provide new molecular evidence for the mechanisms underlying ZXC action.

## Introduction

Ischemic stroke, a leading cause of mortality and morbidity worldwide (Feigin et al., 2003), has become the most common cause of death in China (Zhang et al., 2003). However, the current options for the effective treatment of ischemic stroke remain limited (Truelsen et al., 2007; Mozaffarian et al., 2015). Only 2–5% of stroke patients benefit from thrombolytic therapy to restore blood flow because of the narrow post-stroke time window (<4.5 h), in which ischemic tissue can be rescued (Hacke et al., 2008; Del Zoppo et al., 2009). Thus, the effect of interventional neuroendovascular therapies is still under evaluation (Chimowitz, 2013; McTaggart et al., 2015).

Basic research studies in animals are conducted to investigate the pathological changes that occur after stroke and explore the efficacy of new drugs and therapies. Brain anoxia and ischemia resulting from ischemic stroke trigger the necrosis and apoptosis of nerve cells, which are important contributors to the damage caused by cerebrovascular disease. Blocked cerebral blood vessels can be recanalized by various techniques that redirect blood supply back to ischemic brain tissue, thereby improving the cerebral ischemia and decreasing nerve cell apoptosis for a brief period. However, reperfusion injuries occur, and neurological function cannot be restored. The focal middle cerebral artery occlusion (MCAO) model, representative of the reperfusion of human ischemia caused by stroke, is used to study the pathological changes and mechanisms of drug action following ischemic stroke (Longa et al., 1989; Belayev et al., 1996a; Belayev et al., 1996b). Cell death after MCAO stroke occurs due to complex interactions among excitatory amino acid (EAA) toxicity, acidosis, inflammatory responses, oxidative stress, peri-infarct depolarization, and apoptosis (Nygren et al., 2006; Zheng et al., 2009; Ziemka-Nalecz and Zalewska, 2012). Anoxia and ischemia increase reactive oxygen species (ROS) production in cells (Kaur and Ling, 2009; Qin and Crews, 2012; Yang et al., 2015) and mediate inflammatory responses (Barone et al., 1997; Wang, 2005; Qin and Crews, 2012), thus causing cytotoxicity (Savitz et al., 2000). In hypoxic-ischemic brain tissues, both ROS and inflammatory factors affect the development of ischemic pathology, and high concentrations of ROS damage cell components, hence affecting cell growth (Geng, 2015).

Zhenlong Xingnao Capsule (ZXC) is a classic Tibetan medicine created by Tibetan doctor Sheng Yu Tuo-Yuan Dan Gong Bu in the 8th century A.D. It was first recorded in the Four Medical Tantras and was prescribed for treating cerebrovascular diseases. Now, ZXC is produced by modern extraction and separation technology. Its main functions include inducing resuscitation, clearing heat, dredging meridians, and treating stroke caused by phlegm and blood stasis, dysphasia, hemiplegia, and facial paralysis. ZXC has obvious therapeutic effects on ischemic brain injury and cerebral apoplexy and its sequelae with few side effects, and it is widely used by the Tibetan population. However, modern research on this medicine is limited, and systematic investigations of its effects and mechanisms of action are scarce. Therefore, we explored the effects and properties of ZXC in the treatment of cerebral apoplexy induced by hypoxia and aim to provide support for its clinical applications.

## Materials and Methods

### Drugs and Chemicals

Gallic acid (lot number: 110831-200803, purity [mass fraction]: 90.1%), glycyrrhetinic acid (lot number: 110723-200612, purity: 99.5%), licorice glycosides (lot number: 111610-201106, purity: 93.7%), cinnamic acid (lot number: 110786-200503, purity: 99.9%), muscone (lot number: 110719-201215, purity: 99.6%), cinnamic aldehyde (lot number: 110710-201418, purity: 99.4%), crocin I (lot number: 111588-201303, purity: 92.6%), crocin II (lot number: 111589-201103, purity: 91.9%), eugenol (lot number: 110725-201213, purity: 99.7%), and Glu (lot number: 140690-201203, purity: 100%) were purchased from the National Institutes for Food and Drug Control (Beijing, China). Tau (lot number: T103829, purity: 99%), GABA (lot number: A104200, purity: 99%), L-homoserine (lot number: H105430, purity: 98%), ethanethiol (lot number: E110411, purity: 98%), and o-phthalaldehyde (lot number: P108632, purity: 98%) were purchased from Shanghai Aladdin Biochemical Technology Co. Ltd. (Shanghai, China).

Appropriate concentrations of the major effective components of ZXC were prepared in mixed standard samples in 80% methanol according to the linear range of their chromatographic detection. The samples contained 20 μg/ml of gallic acid, 2.04 μg/ml of cinnamic acid, 1.86 μg/ml of liquiritin, 2.2 μg/ml of crocin I, 2.0 μg/ml of crocin II, 4.2 μg/ml of cinnamaldehyde, 95 μg/ml of eugenol, 0.258 μg/ml of glycyrrhetinic acid, and/or 7.38 μg/ml of muscone.

ZXC, with a specification of 0.3 g/tablet, was produced by Jinke Tibetan Medicine Co., Ltd. (Xining, China). The approval number is Z20026000, and production batch number is 20130110. The main compounds in ZXC are presented in **Table S1**. Negative control samples for ZXC were also provided by Jinke Tibetan Medicine Co., Ltd. The chromatography samples were prepared as follows: 1 g of ZXC was precisely weighed, placed in a conical flask with a cover, and 25 ml of 80% methanol was added. The sample was weighed again, treated with ultrasound (250 W, 45 kHz) for 40 min, and then added to a 30°C water bath. The sample was removed, cooled, and weighed again, and the lost mass was offset with 80% aqueous methanol. The sample was shaken evenly and filtered through a 0.22-μm filter membrane. The primary filtrate was then discarded, and the subsequent filtrate used as the test solution.

Acetonitrile, acetone, methanol, and formic acid, which were all chromatographically pure, were purchased from Sinopharm Chemical Reagent Co., Ltd. (Shanghai, China). We used ultrapure water obtained from a Milli-Q Water Purification System; other common reagents were analytically pure and purchased from Sinopharm Chemical Reagent Co., Ltd.

### Primers, Antibodies, and Kits

The primers used in real-time polymerase chain reaction (RT-PCR) are listed in **Table S2**. The primary antibodies against p38 (#9790), Bax (#14796), NF-кB (#9609), Bcl-2 (#2870), and caspase-3 (#9661) used in the western blots were obtained from Cell Signaling Technology, Inc. (Danvers, MA, USA), while the antibody against beta-actin (ab8227) was purchased from Abcam plc (Cambridge, UK). The recommended dilution ratios of all primary antibodies were applied. The secondary antibody used in the western blots, goat anti-rabbit IgG/HRP (PV-6001), was obtained from ZSGH-Bio (Beijing, China). The primary antibodies used for IHC were the MAPK14 (p38) antibody and CASP3 (P17) antibody; both were obtained from Boster Biological Technology Co., Ltd. (Wuhan, China). A diaminobenzidine detection kit (streptavidin-biotin), which included biotin-goat anti-rabbit IgG, streptavidin-peroxidase, diaminobenzidine substrate, and other reagents, was obtained from Fuzhou Maixin Biotech Co., Ltd. (Fuzhou, China). The hematoxylin and eosin (HE) staining solution used for counterstaining in IHC was also obtained from Fuzhou Maixin Biotech Co., Ltd. The kits used for detecting ROS, MDA, and GSH-Px were purchased from Beyotime Biotechnology Co., Ltd. (Shanghai, China), Beijing Solarbio Science & Technology Co., Ltd. (Beijing, China), and Sigma-Aldrich Corporation (St. Louis, MO, USA), respectively. The kits used for detecting T-AOC and SOD were both from Nanjing Jiancheng Bioengineering Institute (Nanjing, China).

### Animals and Cell Lines

Twenty-four pathogen-free male Wistar rats (weight: 200–250 g) were provided by Beijing Vital River Laboratory Animal Technology Co., Ltd. (Beijing, China; production license number: SCXK[Jing]2012-0001). The rats were divided into eight treatment groups with three rats each, which were labeled according to the length of drug administration: 0 (blank group), 3, 5, 6, 7, 8, 9, or 10 d. These time points were determined using preliminary experiments, in which cinnamic acid was used as the index component.

Pathogen-free male SD rats (n = 205; weight upon receipt: 120–140 g; weight at experiments: 140–160 g) were provided by Beijing Vital River Laboratory Animal Technology Co., Ltd. The breeding environment consisted of a barrier-class animal laboratory (license number: SYXK[Lu]2013-0012) with the following parameters: temperature: 20–25°C, daily fluctuation in temperature: ≤3°C, humidity: 40–70%, air change rate: ≥10 times/h, and lighting time: 12:12 h L/D (lights on at 8:00 am and off at 8:00 pm). The laboratory animals were cared for according to “*The Care and Use of Laboratory Animals*” by the Laboratory Animal Center of Shandong Traditional Chinese Medicine University.

The BV-2 cells were maintained in Dulbecco’s modified Eagle’s medium (GE Healthcare Life Sciences, Logan, UT, USA) with 10% fetal calf serum and incubated at 37°C in 5% CO_2_ until cultivation.

### Ultra-Performance Liquid Chromatography

The UPLC experiments were performed on an Agilent 1290 Infinity LC (Agilent Technologies Inc., Santa Clara, CA, USA). The chromatographic column consisted of the following: Agilent Zorbax C18 (100 × 2.1 mm, 1.8 μm), moving phase A: 0.1% aqueous methanol, and moving phase B: acetonitrile. The gradient elution was 0–6.0 min, 10–30% acetonitrile; 6.0–13.0 min, 30–60% acetonitrile; 13.0–18 min, 60–90% acetonitrile; 18.0–19.5 min, 90% acetonitrile; 19.5–19.7 min, 90–10% acetonitrile; and 19.7–23.0 min, 10% acetonitrile. The sectioned changing wavelength detection was as follows: 274 nm from 0 to 3.0 min (gallic acid), 440 nm from 3.0 to 5.0 min (crocin I, II), 237 nm from 5.0 to 6.0 min (liquiritin), 270 nm from 6.0 to 7.0 min (cinnamic acid), 280 nm from 7.0 to 9.0 min (eugenol, cinnamaldehyde), 254 nm from 9.0 to 13.0 min (glycyrrhetinic acid), and 283 nm from 13.0 to 23.0 min (muscone). The volume flow rate was 0.3 ml/min, column temperature was 35°C, and sample size was 1 μl. The limit of detection was defined as three times the signal-to-noise ratio (SNR), and the limit of quantitation was defined as 10 times the SNR. We used the solvent as a control and compared the response values of the solvent and samples at the same retention times.

### Drug Serum Preparation

In all groups, blood was obtained from the abdominal cardinal veins 0.5, 1, and 2 h after intragastric administration on the corresponding days. In accordance with the preliminary experiments, the optimized ZXC administration dosage was 250 mg·kg^−1^·d^−1^. After standing at 4°C for 30 min, the samples were centrifuged for 10 min at 2,000 × *g*/min, and the supernatant was collected. To 100 μl of supernatant, 400 μl of methanol was added, and the solution was flipped and mixed evenly, extracted after 10 min of ultrasound processing, and centrifuged at 20,400 × *g*/min for 10 min. Subsequently, 420 μl of the supernatant was collected and blow-dried in nitrogen, 200 μl of methanol was then added, and the redissolved samples were again centrifuged at 20,400 × *g*/min for 10 min. Finally, the supernatant was extracted.

### BV-2 Cell Hypoxia Model and ROS Detection

Serum-free medium containing 250 μM of CoCl_2_ was used to cultivate the cells and create a chemical hypoxia model. The medium was divided into serum-administration and soup-cultivation groups. The culture medium contained 10% drug serum in the serum-administration group and a certain concentration of soup in the soup-cultivation group. The complete culture medium containing 10% fetal bovine serum was used in the control group for 24 h of cultivation.

ROS detection was performed in strict accordance with the specifications outlined by the supplier. In brief, following the identification of ROS after adding dichloro-dihydro-fluorescein diacetate, CytoFLEX FCM (Beckman Coulter, Inc., Brea, CA, USA) was used to detect the cell fluorescence intensities in each group using an excitation wavelength of 488 nm and emission wavelength of 525 nm.

### Rat Cerebral Ischemia-Reperfusion Injury Model

The experimental groups comprised a sham group, MCAO group, low-ZXC group, high-ZXC group, and nimodipine group, with 10 rats in each group. Rats from the MCAO group were anesthetized using 10% chloral hydrate at a dosage of 3.5 ml·kg^−1^. Based on a previous study (Clark et al., 1997), the MCAO operation was conducted using a reversible cerebral artery occlusion suture method that was designed for rats, which was followed by stitching and sterilization. The sham group underwent surgery similar to that of the MCAO group, but the external carotid artery and branches were not actually blocked. Both sham and MCAO groups were intragastrically administered 0.5% sodium carboxymethyl cellulose (Alibaba Co., Ltd., Hangzhou, China).

According to the US Food and Drug Administration’s “Guidance for Industry and Reviewers. Estimating the Safe Starting Dose in Clinical Trials for Therapeutics in Adult Healthy Volunteers,” a rat’s human-equivalent dose is 6.2 times that of a human’s clinical dose. Hence, the high dose was set at 250 mg·kg^−1^·d^−1^, which was 12.34 times the clinical dosage (i.e., twice the equivalent human dose). The low dose was set at 125 mg·kg^−1^·d^−1^, which was 6.17 times the human clinical dose. The intragastric administration dose of the nimodipine tablets was 37 mg·kg^−1^·d^−1^, which was similar to the clinical dose. The intragastric administration volume was 10 ml·kg^−1^, with an administration cycle of 14 d.

### Cerebral Ischemia-Reperfusion Injury Model Evaluation

A MoorFLPI-2 speckle flow system (Moor Instruments, Devon, UK), which uses the laser speckle contrast technique to deliver real-time and high-resolution blood flow images, was used to monitor cerebral blood flow occlusion. When blood flow occlusion reached or exceeded 70%, the model was considered successful. Ninety minutes after the ischemia, the suture was taken out for re-reperfusion (Yunoki et al., 2014), and neurological function was evaluated after an additional 90 min. The neurological disorder evaluations were scored according to Longa et al. (1989): a score of 0 indicated no neurological injury symptoms, a score of 1 indicated that the forelimb opposite the focus could not be completely straightened while lifting the tail, a score of 2 indicated that the forelimb opposite the focus buckled while lifting the tail, a score of 3 indicated that the rat whirled slightly to the side of paralysis when walking, a score of 4 indicated that the rat whirled considerably to the side of paralysis when walking, and a score of 5 indicated that the rat could not walk and fell down. The MCAO animals with neurological function scores of 1–2 and sham-operated animals with neurological function scores of 0 were included in this experiment.

### Triphenyltetrazolium Chloride (TTC) Staining

TTC staining was performed immediately following the imaging. Serial 2-mm-thick coronal brain sections were incubated in 2% TTC solution (Sigma-Aldrich Corporation) for 30 min at 37°C and then fixed in 4% formaldehyde solution to increase the contrast between normal and ischemic brain tissues. The stained sections were then digitally scanned for analysis.

The infarcted volume ratio and degree of cerebral edema on the ischemic side in each experimental group were calculated using the following formula:

Volume ratio of cerebral infarction (%) = [area of nonischemic hemisphere (mm^2^) − noninfarction area of ischemic hemisphere (mm^2^)] × thickness (3 mm)/whole brain volume (mm^3^)

Edema degree (%) = [volume of ischemic hemisphere (mm^3^) − volume of nonischemic hemisphere (mm^3^)]/volume of nonischemic hemisphere (mm^3^).

### HE Staining and Nissl Staining

The MCAO operation was performed after the drug administration. After I-90, reperfusion was conducted for 24 h. The abdominal aorta was then clipped, followed by myocardial perfusion with normal saline. After the upper limbs and lung tissues became white in color, the cerebral coronalis was dissected into four parts. The two sections in the middle (cortex and hippocampus) were sliced at regular intervals in a histopathological manner. HE and Nissl stainings were conducted, and the tissue was then examined under a microscope. The standard procedures for HE (Wang et al., 2015) and Nissl stainings (Augustinack et al., 2005) were followed.

### Amino Acid Sample Preparation in the Rat Cortex

The rats in the sham, MCAO, and high-ZXC groups (six rats in each) were decapitated, and their brains were removed at six time points (30, 60, and 90 min after the operation and 30, 90, and 180 min after reperfusion) by quickly stripping the cerebral tissues, cutting off approximately 25 mg of frontal lobe tissue, and adding 1 ml of a methanol:water solution (volume ratio 1:1). Later, the tissues were added to an ice bath with ultrasound for 15 min and then homogenized in a centrifuge for 30 s at 4°C and 13,000 × *g*. A portion of the supernatant (0.5 ml) was removed, and the solution containing the marker L-homoserine was added to reach a volume of 100 μg/L. After 15 min of centrifugation at 13,000 r/min at 4°C, the supernatant was extracted for immediate use in the derivatization reaction.

### High-Performance Liquid Chromatography

The high-performance liquid chromatography experiments were performed on an Agilent 1260 [Agilent Technologies Singapore (International) Pte. Ltd., Singapore] with precolumn derivation. The software version used was ChemStation (Revision) B.04.03 (16). The derivatization reagent consisted of 27 mg of o-phthalaldehyde and 40 μl of ethanethiol dissolved in 5 ml of methanol to which 5 ml of 0.1 mmol/L sodium tetraborate buffer solution was added. Normal concentrations of amino acid liquid or biological samples (40 μl) were added to 20 μl of the prepared derivatization solution. The solution was mixed for 30 s and then allowed to stand for 120 s. The chromatographic column used was an Agilent ZORBAX C18 column (4.6 × 150 mm, 5 μm). Moving phase A consisted of 10 mmol/L of Na_2_HPO_4_.12H_2_O (pH 6.88), and moving phase B consisted of methanol/acetonitrile (3:1). The ratio of phases A and B in solution was 6.4:3.6. The wavelength detected for excitation (λex) was 355 nm, and the emission wavelength (λem) was 450 nm. The gain margin was ×4 with a lower sensitivity. The flow rate was set to 1.0 ml/min, the column temperature was 35°C, and the sample size was 10 μl.

### Antioxidant Index Determination

After the drug administration, the rats in all groups (sham, MCAO model, low-ZXC, high-ZXC, and nimodipine; 10 rats per group) were anesthetized for the MCAO operation using 10% chloral hydrate. The suture was taken out after 90 min of ischemia. After reperfusion was performed for 22 h, the rats were decapitated. The cerebral tissues were kept in cold normal saline to remove blood and then dried with filter paper. The left and right cerebral hemispheres were cut for weighing to prepare a 10% tissue homogenate. The relevant index detection was conducted according to the instructions of the kit, with strict adherence to the kit’s operational requirements and notes. A UV-2550 ultraviolet spectrophotometer (Shimadzu Scientific Instruments, Kyoto, Japan) was used for detection.

### Real-Time Polymerase Chain Reaction

After a cycle of drug administration in all groups (sham, MCAO model, low-ZXC, and high-ZXC; three rats/group/detection time point), the MCAO operation was performed on the rats in the MCAO model, low-ZXC, and high-ZXC groups. All rats were anesthetized using 10% chloral hydrate. For the sham group, no sutures were made, and a right-side cerebral artery occlusion model was prepared for the evaluation. After the MCAO operation, decapitation was performed at of I-30, I-90, I-90 + R-30, and I-90 + R-180. RNA Lyzol (Shanghai ExCell Biology, Inc., Shanghai, China) was used for RNA extraction. A Thermo Scientific RevertAid First Strand cDNA Synthesis Kit (Thermo Fisher Scientific Inc., Waltham, MA, USA) was used for reverse transcription, and 5 μg of RNA was used for each sample. RT-PCR Master Mix (Toyobo Co., Ltd., Osaka, Japan) was used for RT-PCR, which was run on an ABI 7500 fast real-time fluorogenic quantitative RT-PCR system.

### Western Blot and IHC

Western blotting (Kaur and Bachhawat, 2009) and IHC (Tian et al., 2013) were performed according to standard procedures. All reagents and kits were used according to the manufacturer’s instructions. The antibodies were listed in the “Primers, antibodies, and kits” section.

### Statistical Analysis

All data were analyzed with GraphPad Prism 5 statistical software (GraphPad Software, Inc., La Jolla, CA, USA). One-way analyses of variance were performed for the comparisons among the groups. *P* < .05 was considered statistically significant.

## Results

### Concentrations of the Major Effective Components of ZXC

**Figure 1:**
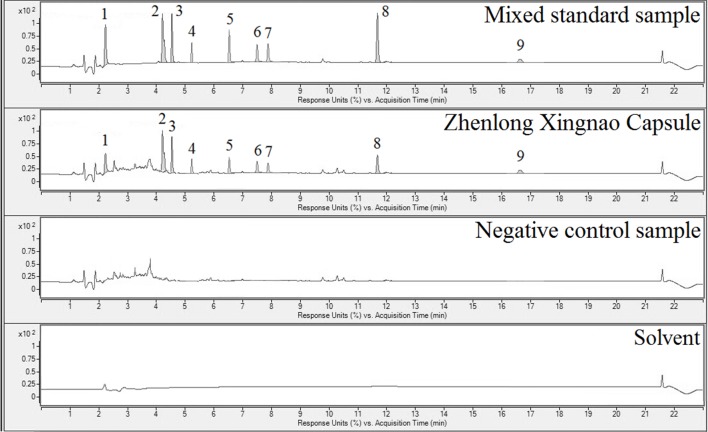
Ultra-performance liquid chromatography (UPLC) results for the mixed reference sample (1^st^ panel), Zhenlong Xingnao Capsule (ZXC) sample (2^nd^ panel), negative control sample (3^rd^ panel), and solvent (4^th^ panel). 1: gallic acid; 2: crocin I; 3: crocin II; 4: liquiritin; 5: cinnamic acid; 6: cinnamaldehyde; 7: eugenol; 8: glycyrrhetinic acid; 9: muscone.

ZXC chromatographic samples were prepared in three batches for ultra-performance liquid chromatography (UPLC; **Figure 1**). Comparisons with mixed check samples were performed, and a one-point external standard method was used to calculate the concentrations. The concentrations of the components in the three batches of ZXC samples are listed in **Table 1**. The mass fractions of gallic acid, crocin I and II, liquiritin, cinnamic acid, cinnamaldehyde, eugenol, glycyrrhetinic acid, and muscone were 0.422–0.448, 0.093–0.105, 0.096–0.112, 0.0268–0.0285, 0.142–0.153, 0.140–0.158, 1.519–1.547, 0.00755–0.00804, and 0.117–0.121 mg/g, respectively. These results showed that the batches had slight differences and were stable.

**Table 1:** Concentrations of the index components of Zhenlong Xingnao Capsule (n = 3).

Lot number	Mass fraction (mg·g^−1^)								
	Gallic acid	Crocin I	Crocin II	Liquiritin	Cinnamic acid	Cinnamaldehyde	Eugenol	Glycyrrhetinic acid	Muscone
20131008	0.422	0.105	0.112	0.0285	0.149	0.140	1.532	0.00761	0.119
20130110	0.448	0.093	0.102	0.0268	0.142	0.145	1.547	0.00755	0.117
20130701	0.434	0.098	0.096	0.0277	0.153	0.158	1.519	0.00804	0.121

### Effects of ZXC Intervention on ROS in a BV-2 Cell Hypoxia Model

**Figure 2:**
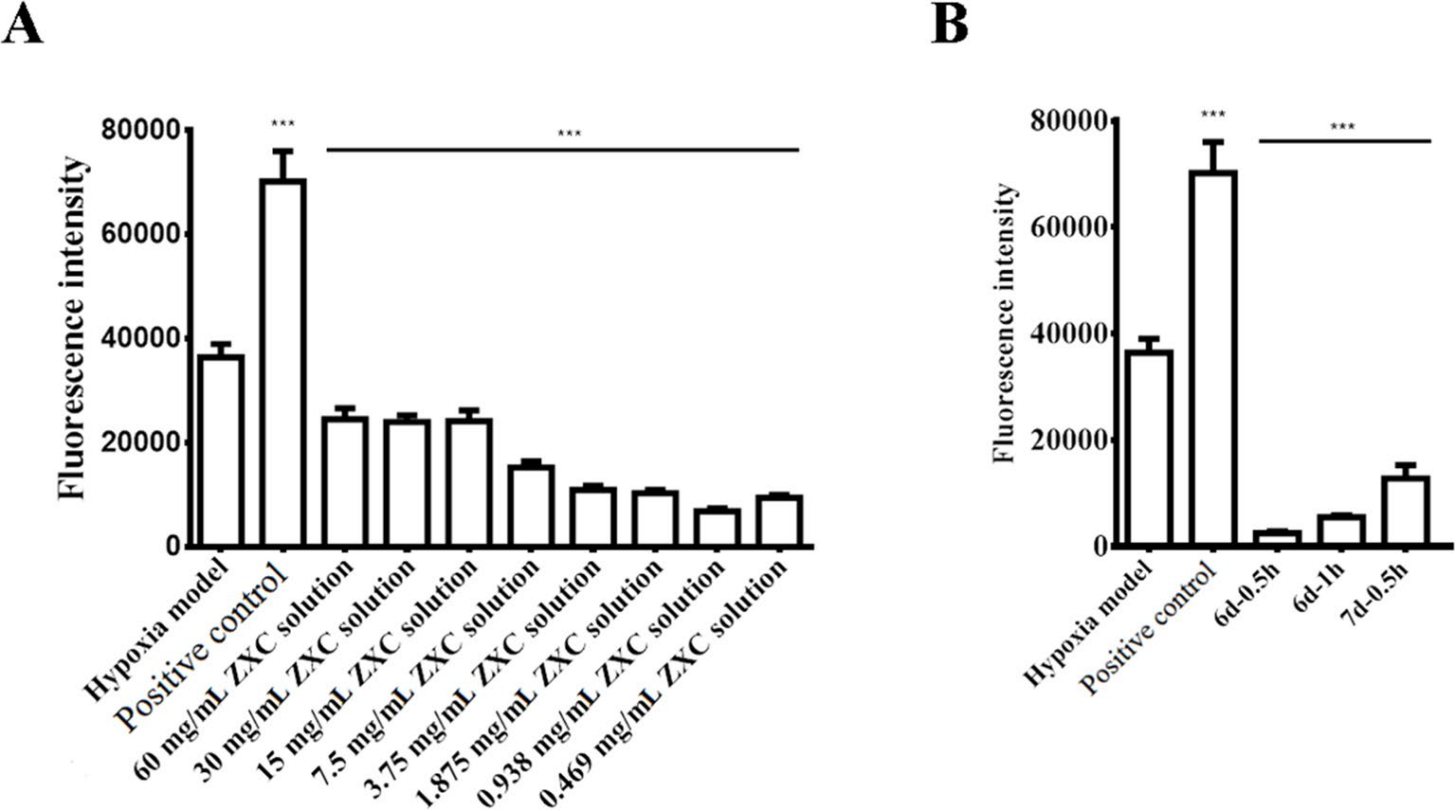
Effects of ZXC on reactive oxygen species (ROS) levels in a BV-2 cell model of hypoxia-regeneration. **(A)** Solutions with different concentrations of ZXC were added directly to BV-2 cells exposed to hypoxia-reoxygenation. **(B)** The effects of the ZXC-containing serum interventions on the effects of hypoxia-reoxygenation in a BV-2 cell model. ***P < 0.001 *vs.* model group; the data are expressed as mean ± standard deviation (SD; n = 4).

We prepared a BV-2 cell hypoxia model *in vitro* using hypoxia-reoxygenation and examined ROS production in the model after intervention with ZXC to evaluate its ability to protect nerve cells. The results showed that ROS production increased after the cessation of BV-2 cell hypoxia (**Figure 2A**). Direct intervention with ZXC solution decreased ROS production, which gradually decreased with increasing concentrations of ZXC, with the 0.938-mg/ml ZXC soup having the largest decrease in ROS production. In the groups treated with 0.469 mg/ml of ZXC, ROS production increased slightly. When the cells were treated for 0.5 h with serum treated with ZXC for 6 days (6 days–0.5 h), 1 h with serum treated with ZXC for 6 days (6 days–1 h), or 0.5 h with serum treated with ZXC for 7 days (7 days–0.5 h), ROS production decreased (**Figure 2B**).

### Protective Effects of ZXC in Rats With MCAO-Induced Cerebral Ischemia-Reperfusion Injury

**Figure 3:**
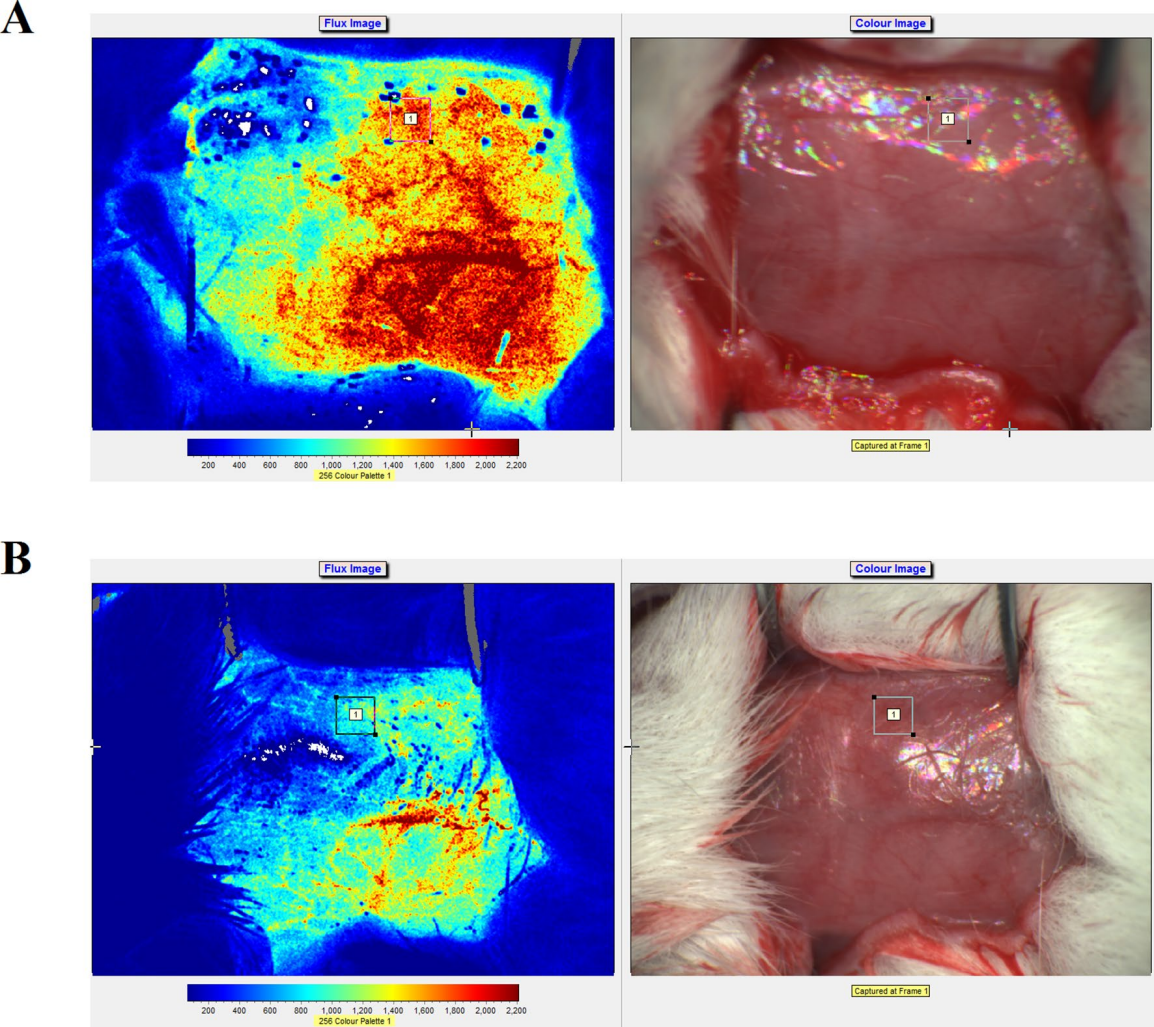
Laser speckle flow imaging results and photographs of the rats before **(A)** and after **(B)** artery ligation on the right side.

Real-time speckle flow imaging revealed that the blood flow occlusion after artery ligation was approximately 78%, which indicated that the model was successfully generated (**Figure 3**). As shown in **Figure 3**, higher signal intensities indicated more abundant blood flow.

Compared with the sham group, rats in the MCAO model group exhibited significant neurological dysfunction. The groups treated with low and high doses of ZXC (low-ZXC and high-ZXC groups) showed significantly decreased neurological function scores compared with those in the MCAO model group (*P* < 0.05). These results revealed that ZXC significantly improved neurological dysfunction caused by cerebral ischemia-reperfusion injury (**Table 2**).

**Table 2:** Effects of Zhenlong Xingnao Capsule (ZXC) on neurological function, volume ratio of cerebral infarction, and degree of edema in rats with cerebral ischemia-reperfusion injury (mean ± standard deviation, n = 10).

Group	Dosage (mg·kg^−1^)	Neurological function score	Volume ratio of cerebral infarction (%)	Degree of edema (%)
Sham		0	0	0
Model		2.08 ± 0.67**	23.02 ± 3.20**	27.22 ± 4.12**
Low ZXC	125	1.42 ± 0.66^##^	14.37 ± 4.24^##^	19.88 ± 3.49^##^
High ZXC	250	1.50 ± 0.67^#^	13.55 ± 1.98^##^	25.00 ± 4.56
Nimodipine	37	1.58 ± 0.52^##^	11.76 ± 3.37^##^	14.33 ± 3.02^##^

**P < 0.01 vs. sham group; ^#^P < 0.05 vs. model group; ^##^P < 0.01 vs. model group.

**Figure 4:**
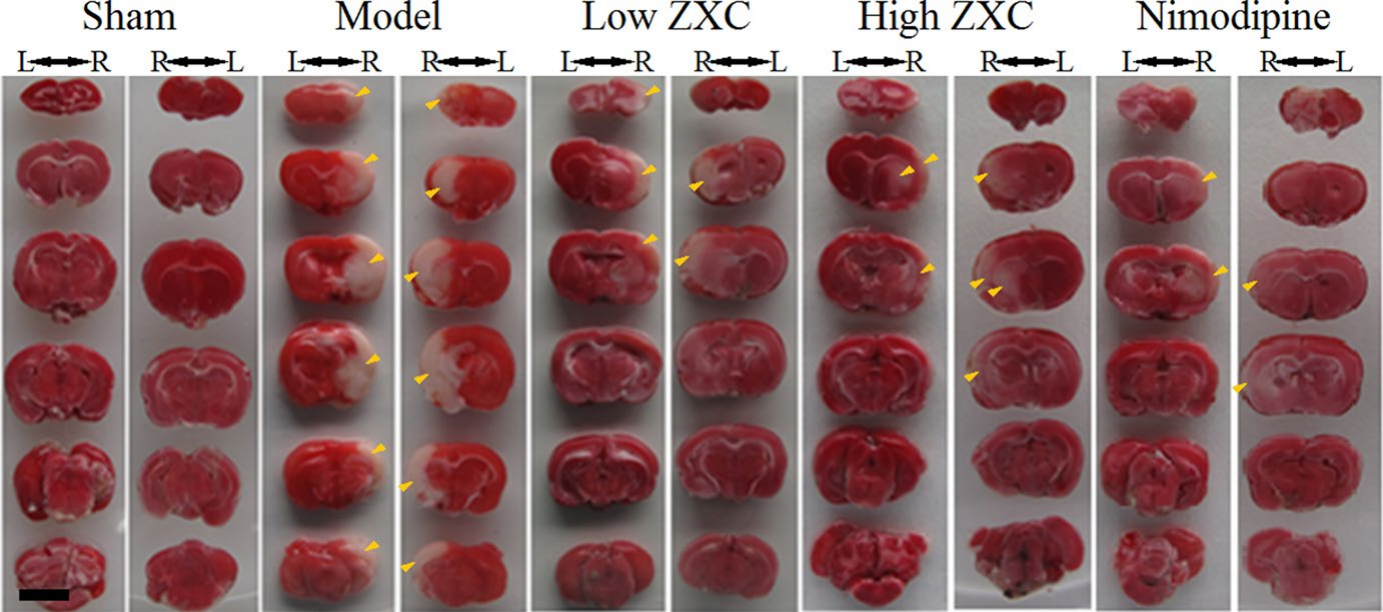
Triphenyltetrazolium chloride staining results of the preventive administration of Zhenlong Xingnao Capsule (ZXC) [left (L) and right (R); back view of rats]. Scale bar: 5 mm. Yellow arrowheads: ischemic tissue (white in appearance).

The protective effects of nimodipine and its molecular mechanisms in the treatment of ischemic neuronal damage are well established (Nuglisch et al., 1990). Here, we used nimodipine as a positive control. Compared with the sham group, significant areas of ischemia-related necrosis and edema were observed in the MCAO model group. After staining the tissue with triphenyltetrazolium chloride (TTC), healthy brain tissues appeared deep red while ischemic tissues appeared white. The TTC staining results are shown in **Figure 4**. Compared with the MCAO model group, the low- and high-ZXC and nimodipine groups showed significant decreases in their cerebral infarcted volume ratios (*P* < 0.01). Compared with the MCAO model group, the low-ZXC and nimodipine groups showed significantly decreased degrees of edema (*P* < 0.01; **Table 2**)

The cortices of the rats in the MCAO group exhibited disordered nerve cells, partial nerve cells with significant mutations and necrosis, cell swelling, inflammatory cell infiltration, interstitial edema, and colloid nodules. The nerve cells of rats in the low- and high-ZXC and nimodipine groups were arranged in an orderly manner compared with those in the MCAO model group. In addition, these groups showed less interstitial edema and mutated and necrotic nerve cells. The arrangement of the hippocampal pyramidal cells in the MCAO model rats was disorderly, with partial cells exhibiting mutations and necrosis as well as swelling. The pyramidal cells in the low- and high-ZXC and nimodipine groups were arranged in a more orderly manner than those in the MCAO model group, and they had significantly less edema (**Figure S1A**).

Nissl staining is a method used to examine histopathological changes in the Nissl bodies in neuronal cytoplasm and neuronal injuries. The Nissl staining results revealed that the tiger spot-shaped Nissl bodies in the hippocampal CA1 neurons in the MCAO group were sparsely arranged and reduced in volume and number and in states of fragmentation and dissolution. In the nimodipine and low- and high-ZXC groups, the Nissl bodies in CA1 were densely arranged and increased in number, with large volumes compared with those in the MCAO model group. In the MCAO group, the number and volume of the cortical neurons were decreased, the spaces around the Nissl bodies were enlarged, and the neurons were sparsely arranged. Compared with the MCAO group, the nimodipine and low- and high-ZXC groups exhibited significant increases in the cortical neuron number and volume, decreased space around the Nissl bodies, and densely arranged neurons (**Figure S1B**).

### Effects of ZXC on Cortical Amino Acids in the Rats With MCAO-Induced Cerebral Ischemia-Reperfusion Injury

**Figure 5:**
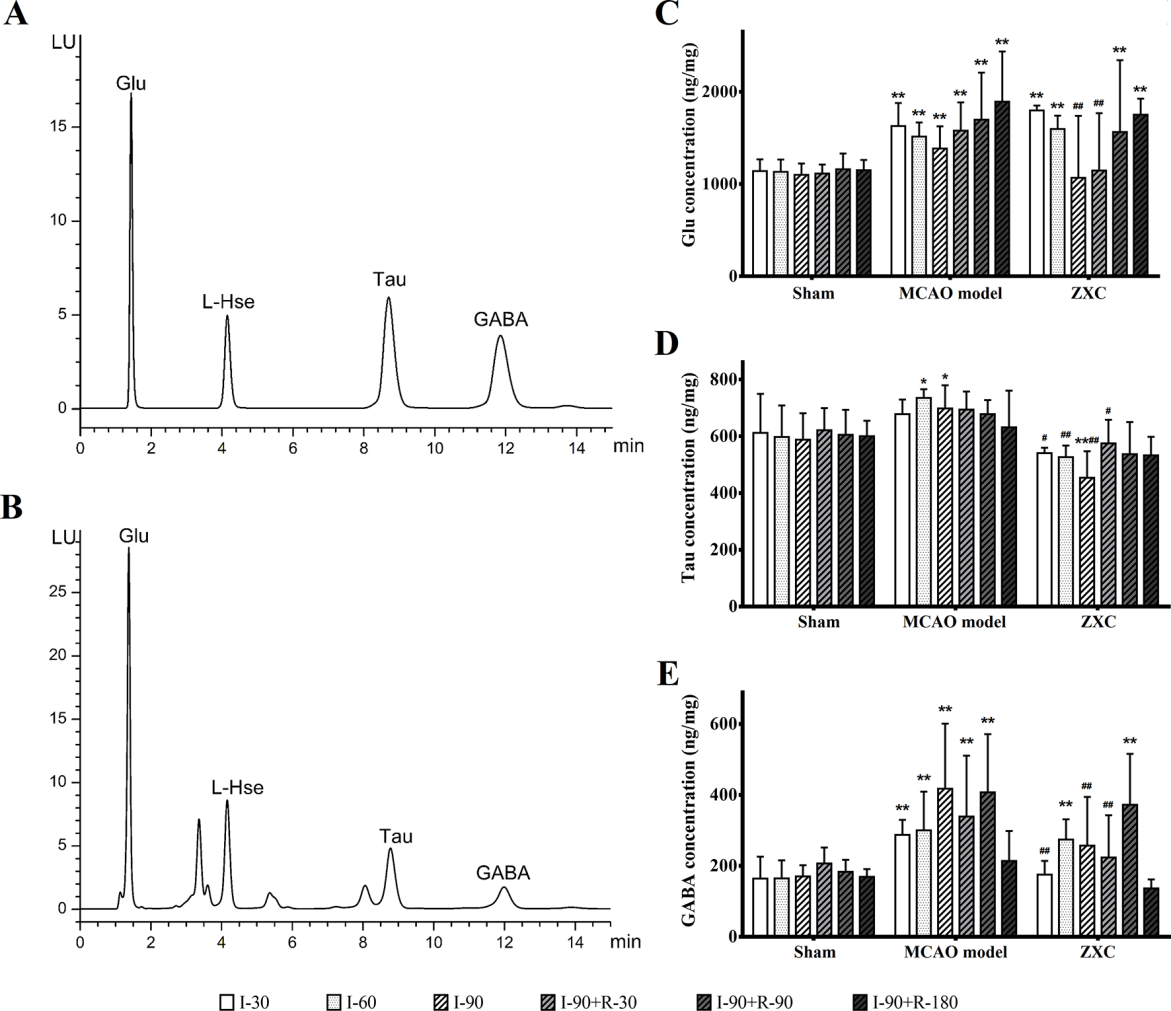
**(A**–**B)** Chromatogram of the mixed standard **(A)** and rat tissue **(B)** samples. **(C**–**E)** The changes in glutamic acid (Glu; **C**), taurine (Tau; **D**), and γ-aminobutyric acid (GABA; **E**) concentrations in the frontal cortex in middle cerebral artery occlusion (MCAO) model rats after Zhenlong Xingnao Capsule (ZXC) intervention for ischemia and reperfusion (ng·mg^−1^, mean ± standard deviation; n = 6). *P < 0.05 *vs.* sham group; **P < 0.01 *vs.* sham group; ^#^*P* < 0.05 *vs.* model group; ^##^*P* < 0.01 *vs.* model group. L-Hse, L-homoserine.

Three amino acids and homoserines were detected with chromatography and separated within 15 min, and their retention times were 1.406 min (Glu), 9.700 min (Tau), and 13.200 min (GABA) (**Figures 5A, B**).

Compared with the sham group, the Glu concentrations in the MCAO group were significantly increased at each time point of cerebral ischemia and reperfusion (*P* < 0.01). Compared with the sham group, the Glu concentrations in the ZXC group were significantly increased after 90 and 180 min of reperfusion (R-90 and R-180, respectively) and after 30 and 60 min of ischemia (I030 and I-60, respectively; *P* < 0.01). After 90 min of ischemia (I-90) and 30 min of reperfusion (R-30), the Glu concentrations returned to normal levels. Compared with the MCAO group, the Glu concentrations in the ZXC group were decreased at I-90 and R-30 (*P* < 0.01). The results are presented in **Figure 5C**.

Compared with the sham group, the tau concentrations in the MCAO group were significantly increased at I-60 and I-90 (*P* < 0.05). At I-90, the concentrations were decreased in the ZXC group (*P* < 0.05). Compared with the MCAO group, the tau concentrations were decreased at I-30, I-60, and I-90 and R-30 in the ZXC treated group. The results are presented in **Figure 5D**.

Compared with the sham group, the GABA concentrations in the MCAO group were significantly increased at I-30, I-60, and I-90 and R-30 and R-90 (*P* < 0.01) while the concentrations in the ZXC group were significantly increased at I-60 and R-90 (*P* < 0.01). Compared with the MCAO group, the GABA concentrations at I-30 and I-90 and R-30 were decreased significantly (*P* < 0.01) in the ZXC group. The results are presented in **Figure 5E**.

### Effects of ZXC on Antioxidant Indices in MCAO-Induced Cerebral Ischemia-Reperfusion Injury

None of the four antioxidant indices were significantly changed in the left-brain tissue of the rats in the MCAO model or drug-administered groups compared with the sham group (**Table S3**). Malondialdehyde (MDA) levels were significantly increased in the right-brain (ischemia side) tissue in the model group, while glutathione peroxidase (GSH-Px), total antioxidant capacity (T-AOC), and total superoxide dismutase (T-SOD) levels were significantly decreased (*P* < 0.05, 0.01). Compared with the MCAO model group, the T-AOC and T-SOD activities were significantly increased in the right-brain tissue of the high-ZXC group (*P* < 0.05; **Table S4**).

### Effects of ZXC on P38, NF-κB, Bcl-2, Bax, and Caspase-3 Expressions in the Frontal Lobe of Rats With MCAO-Induced Cerebral Ischemia-Reperfusion Injury

**Figure 6:**
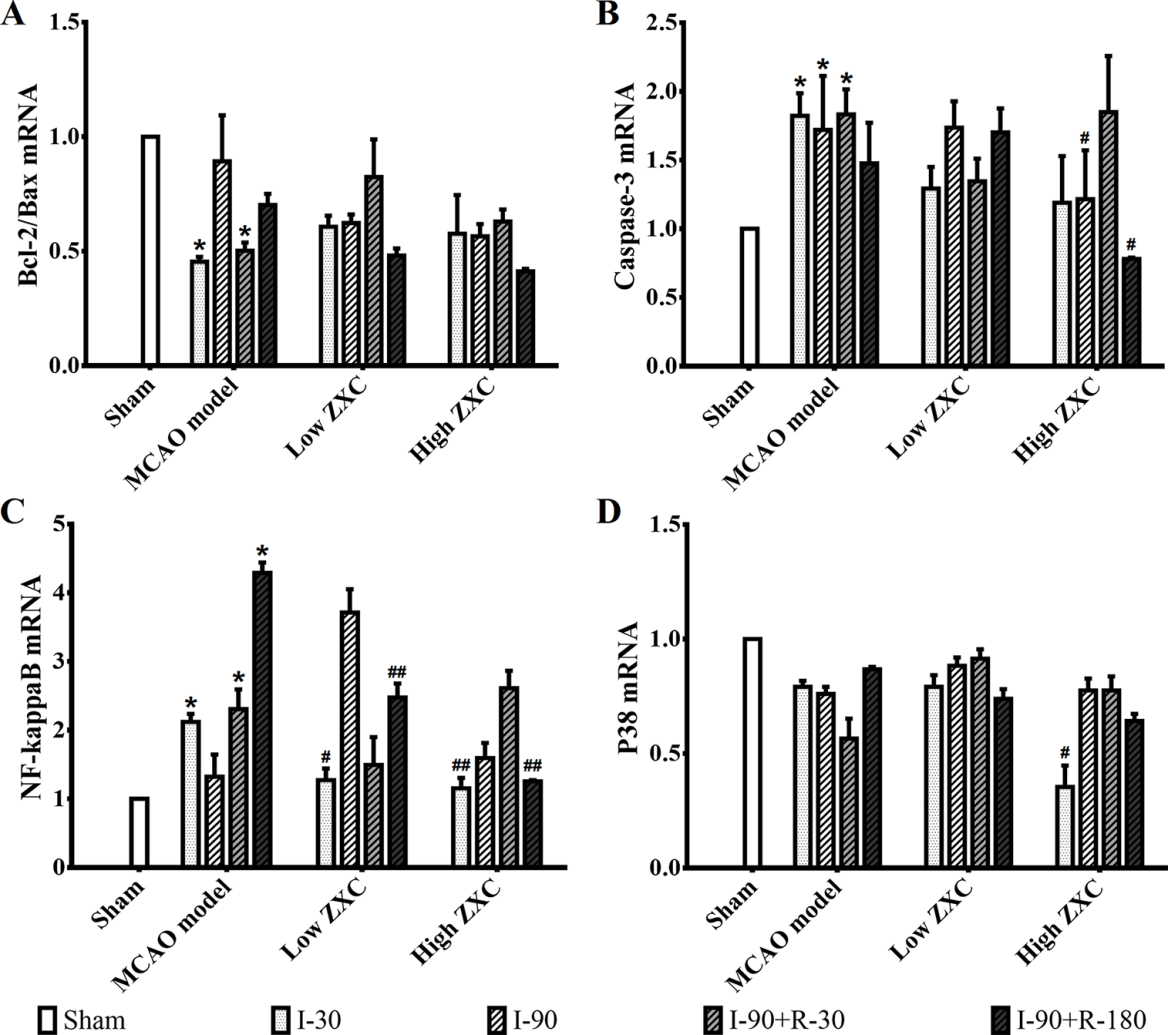
Effects of ZXC on the mRNA levels of the Bcl-2/Bax ratio **(A)**, caspase-3 **(B)**, nuclear factor (NF)-кB **(C)**, and p38 **(D)** in the prefrontal cortex of ischemia-reperfusion injury rats. The data are expressed as mean ± standard deviation (n = 3). I-30, ischemia for 30 min; I-90, ischemia for 90 min; I-90+R-30, ischemia for 90 min, then reperfusion for 30 min; I-90+R-180, ischemia for 90 min, then reperfusion for 180 min. *P < 0.05 *vs.* sham group; ^#^*P* < 0.05 *vs.* model group; ^##^*P* < 0.01 *vs.* model group.

The histogram of the mRNA expression results shown in **Figure 6** indicates that the Bcl-2/Bax ratio was significantly decreased in the MCAO model group compared with the sham group at the I-30 and I-90 + R-30 time points. Compared with the MCAO model group, the ratio was not upregulated in the low- and high-ZXC groups, and the p38 mRNA expression levels were unchanged. The caspase-3 and NF-кB mRNA expression levels were significantly higher in the model group than in the sham group. In the high-ZXC group, caspase-3 expression was downregulated, with significant differences at I-90 and I-90 + R-180. In the low- and high-ZXC groups, NF-кB mRNA expression was downregulated at certain time points of ischemia and reperfusion, with significant differences at I-30 and I-90 + R-180.

**Figure 7:**
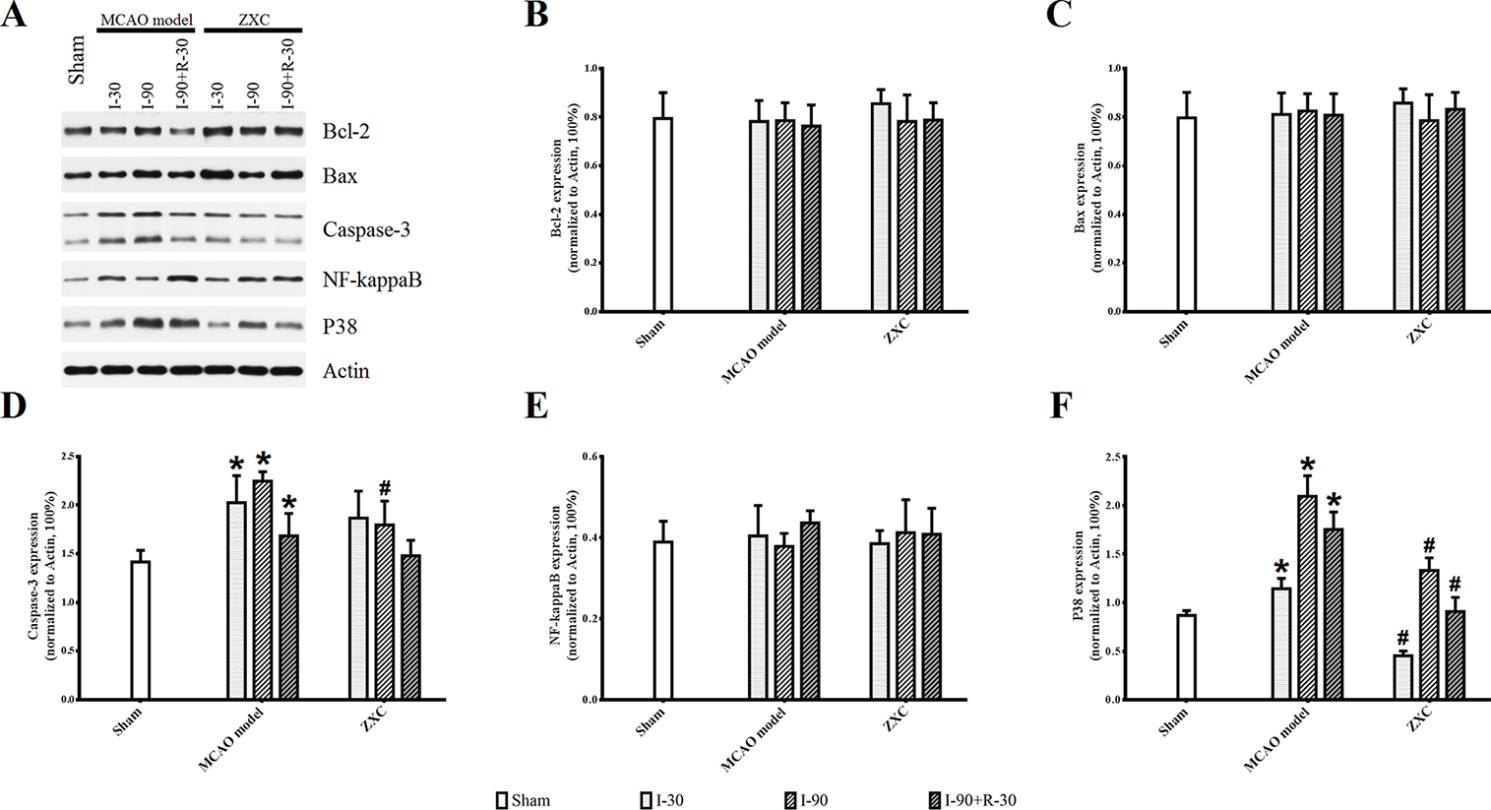
Western blotting results for Sham, Model and ZXC groups **(A)**. Effects of ZXC on the protein expressions Bcl-2 **(B)**, Bax **(C)**, Caspase-3 **(D)**, NF-кB **(E)**, and p38 **(F)** in brain tissue of ischemia-reperfusion injury rats induced by MCAO. The data are expressed as mean ± standard deviation (n = 4). I-30, ischemia for 30 min; I-90, ischemia for 90 min; I-90+R-30, ischemia for 90 min, then reperfusion for 30 min. *P < 0.05 *vs.* sham group; ^#^P < 0.05 *vs.* model group.

Compared with the sham group, p38 and caspase-3 expressions in the frontal cortex were increased at different time points of ischemia in the MCAO model group, while no significant changes were found in NF-кB, Bax, or Bcl-2 expression. Compared with the MCAO model group, p38 protein expression was significantly decreased at different time points in the high-ZXC group (*P* < 0.05), with obvious downregulation in caspase-3 expression at I-90. No obvious changes in the expressions of NF-кB, Bax, or Bcl-2 were found, as presented in **Figure 7**.

Immunohistochemistry (IHC; **Figure S2**, orange/brown cells) showed that the expressions of caspase-3 and p38 were increased at I-90 in the MCAO model group compared with the sham group. The expression of both proteins was lower at I-90 in the ZXC treatment group compared with the MCAO model group, which was consistent with the results of the western blot experiment.

## Discussion

### Major Effective Components of ZXC

A previous clinical study reported that ZXC has explicit effects in cerebral infarction (Ma et al., 2012). The main effective components of this product include gallic acid, cinnamic acid, liquiritin, crocin I, crocin II, cinnamaldehyde, eugenol, glycyrrhetinic acid, and muscone (**Table S1**). This experiment used UPLC and gradient elutions with programmed wavelength change detection to simultaneously quantify the nine index components of ZXC (**Figure 1** and **Table 1**). This is the first study on ZXC, and the results provide important data for further studies on the mechanisms of action of ZXC in the treatment of cerebral infarction.

### ZXC Exhibited Neuroprotection in Two Hypoxia-Reoxygenation Models

In 1987, Iwama et al. (1987) investigated a novel test of drug serum pharmacology and concluded that compound drug serums mainly consist of the active ingredients and their metabolites as well as some impurities. The key factors that influence the pharmacological activity of compound drug serums include the animal species, drug administration cycle, and blood sampling time. Hence, in accordance with the different experimental objectives and systems, preliminary experiments are required to determine the optimal blood sampling time. However, this method is limited by poor repeatability and reliability.

Necrosis and apoptosis resulting from tissue ischemia and hypoxia are important contributors to cerebrovascular diseases and injuries. Cerebral nerve cells include neurons, astrocytes, and microglial cells, and microglial cells are always the target in the treatment of neuroinflammatory and neurodegenerative diseases. ROS generation in hypoxic BV-2 cells has been reported to result in significant increases in their cell volume (Hou et al., 2004; Huang et al., 2005). This study aimed to explore the effects of ROS generation on volume in a BV-2 cell injury hypoxia-reoxygenation model after intervention with ZXC to evaluate the protective role of ZXC on neurocytes (**Figure 2**). Although previous experiments have shown that the cinnamic acid concentrations were the highest at the 7 d–0.5 h time point (data not shown), the ROS generation volume was the lowest after drug intervention. These results suggested no dose-effect relationship between each drug component and the compound preparation and that the effects of the compound preparation were a result of an interaction among the various herbal medicines in the formula. The statistical results and scatter diagrams of the fluorescent results for the direct soup intervention group showed that, for the *in vitro* compound preparations, a higher concentration did not necessarily cause a larger effect. The effects of the soup intervention on the cells included the expected effects, but physical injury was observed when the concentration was too high. Therefore, the intervention effects were only achievable within a certain concentration range (**Figure 2**).

Ischemic cerebral apoplexy is a condition characterized by dysneuria, including hemiplegia and aphasia. Its pathomechanisms manifest in disturbances of cerebral blood supply, tissue hypoxia, ischemic necrosis, and/or encephalomalacia, which results in the swelling of the gray matter and unclear white matter division in the central ischemic area. Tissue stained with terminal deoxynucleotidyl transferase dUTP nick-end labeling exhibits neuron crenation, anachromasis, inflammatory cell infiltration, and gliocyte damage (de Torres et al., 1997; Phanithi et al., 2000; Kundrotiene et al., 2004; Ju and Kim, 2011). Dissolving thrombi to reconnect occluded arteries, recover blood flow, and thereby improve dysneuria symptoms is the only approved regimen for the treatment of acute stage cerebral ischemia (Mehrpour et al., 2013; Emiru et al., 2014). Even if blood flow can be recovered in a timely manner and tissue metabolism improved, only penumbra tissues with functional changes but without necrosis around the infarction site will be saved. Thrombolysis improves prognosis, and the optimal treatment time window is 4–5 h after ischemia. Delayed reperfusion during intra-arterial treatment negatively affects treatment prognosis (Fransen et al., 2016).

MCAO has been widely used to study the pathological changes of apoplexy and the pharmacological mechanisms of drug treatment. Koizumi et al. (1986) established the earliest suture-occluded MCAO model, which was later modified and promoted by Longa et al. (1989) and Aspey et al. (2000), who conducted MCAO operations in three types of rats [Wistar, Sprague–Dawley (SD), and Fisher-344] and found differences in the improvements of movement function, infarction degree, and death rate among the different strains, which suggested differences in their sensitivities to the MCAO operation. Of the different strains of rats, SD rats have fewer mutations in the MCA and good cerebral infarction volume consistency after MCAO. Therefore, we used SD rats in our MCAO model (Kuge et al., 1995; Durukan and Tatlisumak, 2007). At present, the methods for MCAO model evaluation include the dysneuria scoring method by Longa et al. (1989) and determination of cerebral infarction volume *via* TTC staining. Neurological function evaluations must be conducted in drug-free animals, and because of individual differences, tardive neuronal death, and individual subjective judgment, the experimental error is large. However, because this method is noninvasive and allows repeated observations of ischemia and reperfusion at different time points, it is still widely used for MCAO model evaluation. TTC staining of the cerebral infarction volume is considered the gold standard for evaluating models due to its objectivity and accuracy. However, because the use of TTC staining observations cannot continue after the animal is sacrificed and it is extremely difficult to use stained samples to measure other indices, its application is limited. The present study used an MCAO-induced cerebral ischemia-reperfusion injury rat model (**Figure 3**) to examine the protective role of ZXC in cerebral ischemia-reperfusion injury by evaluating neurological function, encephaledema degree, cerebral infarction volume, and injury conditions of the nerve cells and neurons. The results revealed that ZXC significantly improved dysneuria after cerebral ischemia-reperfusion injury, reduced the degree of encephaledema, decreased the cerebral infarction volume ratio (**Figure 4** and **Table 2**), and improved nerve cell function and quantity, indicating that this drug offered protection against cerebral ischemia-reperfusion injury, which was consistent with the experimental results obtained using the *in vitro* hypoxia BV-2 cell model.

Although ZXC is a complex system, benefits for ischemia/reperfusion (I/R) of its major active ingredients have been reported. As a bioactive compound, gallic acid prevented rats from hepatic ischemia and reperfusion injury (Bayramoglu et al., 2015; Ramezani-Aliakbari et al., 2017). Glycyrrhetinic acid could decrease histopathologyical damage and apoptosis in brain tissue injured by I/R damage in C57BL/J6 mouse (Oztanir et al., 2014) and play neuroprotective effects in PC12 cells (Tzu-Chien et al., 2009). One study reported that liquiritin protected against I/R *via* its antioxidant and antiapoptosis properties (Ya-Xuan et al., 2010). Anti-acute myocardial ischemia and anti-cerebral ischemia effect of cinnamic acid have also been studied (Song et al., 2013; Abedin et al., 2014). Eugenol, as known an anti-inflammatory and antioxidant agent, has been widely used in complementary and alternative medicine (Barboza et al., 2018).

### Effects of ZXC on EAA/IAA Homeostasis and Antioxidant Indices in the MCAO Model

Glu is one of the most important EAAs in the brain. The neurovirulent properties of EAAs result in the initiation and execution of cerebral tissue injury (Koizumi et al., 1986). Tau and GABA are important IAAs in the brain; in cerebral ischemia, they release virulence factors that withstand EAAs (Jung et al., 2006; Han et al., 2012). The present study was conducted to assess the neuroprotective mechanisms of ZXC by measuring changes in amino acid concentrations in the cerebral cortex at different time points after ischemia-reperfusion injury and ZXC administration. The Glu/GABA ratio can be used to examine the balance of cerebral EAAs and IAAs. The results of these experiments showed that, in ischemia-reperfusion injury, *in vivo* Glu and GABA levels were increased (**Figures 5C, E**), as were the corresponding overall EAA/IAA balances. In the ZXC prevention group, the concentrations of certain amino acids that are involved in EAA/IAA homeostasis were downregulated at different time points, and they had a tendency to approach normal levels. These results suggested that ZXC exhibited a protective role in the acute stage of ischemia-reperfusion by reducing the acute neurovirulence injuries in cerebral cells that were caused by EAAs. As described in earlier reports (Han et al., 2012), the concentrations of the IAAs tau and GABA were significantly decreased (**Figures 5D, E**), and the Glu/GABA ratio was maintained within homeostasis levels. Maintaining and improving the relative homeostasis of EAA and IAA could be one of the mechanisms underlying ZXC action in reducing the cerebral infarction volume in acute cerebral ischemia. The concentration changes that occur in EAAs and IAAs later in the different treatment groups later in reperfusion, that is, at the clinical cerebral apoplexy sequelae stage, need further studies. The results of this experiment dynamically reflected the concentrations and concentration changes of the three amino acids in the frontal cortex of MCAO rats at different time points of ischemia-reperfusion, hence providing a hypothesized mechanism of ZXC action and a foundation for clinical administration.

The T-AOC of an organism, which reflects the organism’s ability to resist oxidation and scavenge free radicals (Dai et al., 2005), consists of enzymatic and nonenzymatic antioxidant defense systems that include, but are not limited to SOD, catalase, GSH-Px, vitamin C, vitamin E, glutathione, glucose, and β-carotene. The T-AOC, which is used to evaluate whether stress has caused oxidative damage to an organism, is more dependable than information provided by a singular antioxidant index. When endogenous or exogenous stimulation causes abnormalities in an organism’s metabolism and leads to the sudden production of a large number of active ROS, the antioxidant defense system will be triggered and excessive ROS will be removed, thereby protecting the tissues from oxidative damage (Mathew et al., 2007). T-SOD is an endogenous antioxidant enzyme that metabolizes ROS into hypotoxic substances, hence protecting cells from damage and playing a crucial role in balancing oxidation and antioxidation in an organism (Gulesserian et al., 2001; Li et al., 2013). Nonenzymatic reactions trigger lipid peroxidation by attacking polyunsaturated fatty acids in biological membranes and forming lipid peroxides, such as MDA. The MDA volume can therefore reflect the degree of lipid peroxidation and, indirectly, the degree of cell damage. The current study examined the antioxidation mechanisms of ZXC in the protection against ischemic cerebral injury and its influence on antioxidant indices in bilateral cerebral ischemia and nonischemic rat tissues. The results revealed that ZXC significantly improved SOD and T-AOC activities in bilateral ischemic cerebral tissues (**Tables S1** and **S2**), suggesting that *in vivo* antioxidant enzyme synthesis breaks the oxidation/antioxidation balance in stress injury and that ZXC plays an antioxidative role through the joint efforts of enzymatic and nonenzymatic antioxidant defense systems to protect cells from oxidation damage. Nimodipine, which is a calcium ion antagonist that provides protection from cerebral injury, is commonly used as a positive control drug in cerebral ischemia-reperfusion injury experiments. However, in this experiment, no changes were observed in the antioxidant indices in the nimodipine group, suggesting that its cerebral protective role was not realized through antioxidation. Antioxidation may be one of the mechanisms by which ZXC provides protection against cerebral ischemia-reperfusion injury (Nazam Ansari et al., 2008; Zhang et al., 2011).

### Effects of ZXC on Apoptosis-Related Proteins

This study also showed that, when ZXC was used to intervene in MCAO-induced ischemia-reperfusion injury in rats, the expressions of caspase-3 and NF-кB mRNA were downregulated at certain time points of ischemia reperfusion (**Figure 6**). These results suggested that ZXC plays a protective role in ischemia-reperfusion injury through the downregulation of factors associated with apoptosis. Therefore, we studied the role of partial apoptosis correlation factors in acute cerebral ischemia-reperfusion injury by monitoring protein levels to further reveal the changes in cell processes after ZXC intervention. The results revealed a significant increase in p38 protein levels at various time points in the MCAO model and decreased levels in the ZXC prevention group. Caspase-3 expression was downregulated at I-90 in the ZXC prevention group compared with the MCAO model group (**Figure 7** and **Figure S2**), which suggested that ZXC exerts its anti-apoptotic effects by influencing the CD95/Fas signaling pathways. However, this assumption requires further support from additional experiments.

## Conclusion and Future Research

In general, we found that ZXC exerted explicit protective effects against *in vitro* and *in vivo* ischemic and anoxic injuries. A limitation of the current study was that it did not provide a precise understanding of the mechanisms of ZXC. However, the descriptive data still contribute to the understanding of the mechanistic functions of ZXC. The details of the mechanism by which ZXC decreases ROS and inhibits inflammatory factors remain to be elucidated.

## Data Availability

The raw data supporting the conclusions of this manuscript will be made available by the authors, without undue reservation, to any qualified researcher.

## Ethics Statement

This study was carried out in accordance with the recommendations of the Guide for the Care and Use of Laboratory Animals, formulated by the National Institutes of Health, United States. The protocol was approved by the Institutional Committee for Animal Care and Use of Shandong University of Traditional Chinese Medicine.

## Author Contributions

XW, JW, MQ, DH, YW and PS conceived and designed the experiments. XW, QZ, NL, LX, CS, YZ and ZF performed the experiments. SW and YW analyzed the data. CS, YZ, and YW contributed the reagents/materials/analytical tools. XW and PS drafted the manuscript.

## Funding

This study was supported by the National Natural Science Foundation of China (NSFC, Nos. 81874419, 81673719 and 81303074), Ministry of Science and Technology (No. 2017ZX09301064 and 2017ZX09301064002), The Open Project of Key Laboratory of Prevention and treatment of cardiovascular and cerebrovascular diseases, Ministry of Education (No. XN201814), Basic scientific research service fee of Shandong University (NO. 2014QLKY35) and the Shandong Traditional Chinese Medicine Science and Technology Development Program (Nos. 2015-161 and 2013-010).

## Conflict of Interest Statement

The authors declare that the research was conducted in the absence of any commercial or financial relationships that could be construed as a potential conflict of interest.

## Abbreviations

MCA, middle cerebral artery; MCAO, middle cerebral artery occlusion; ROS, reactive oxygen species; EAA, excitatory amino acid; Glu, glutamic acid; Tau, taurine; GABA, γ-aminobutyric acid; IAA, inhibitory amino acid; MAPK, mitogen-activated protein kinase; NF-кB, nuclear factor-кB; ZXC, Zhenlong Xingnao Capsule; UPLC, ultra-performance liquid chromatography; TTC, triphenyltetrazolium chloride; MDA, malondialdehyde; GSH-Px, glutathione peroxidase; T-AOC, total antioxidant capacity; T-SOD, total superoxide dismutase; HE, hematoxylin and eosin; RT-PCR, real-time polymerase chain reaction.
