# Supplementary material for: Neuroprotective Effects and Mechanisms of Zhenlong Xingnao Capsule in In Vivo and In Vitro Models of Hypoxia
Source: Front Pharmacol. 2019 Sep 26;10:1096. doi: 10.3389/fphar.2019.01096 (PMC6775503; doi:10.3389/fphar.2019.01096)

**Supplementary figures**

*Figure S1*

Images of tissues stained with hematoxylin and eosin (A) and Nissl stain (B) from the cortex (top row) and hippocampus (bottom row). The following groups were examined: sham, middle cerebral artery occlusion (MCAO) model, low-Zhenlong Xingnao Capsule (ZXC), high-ZXC, and nimodipine. Scale bar: 50 μm. The magnification of all samples shown here is the same.


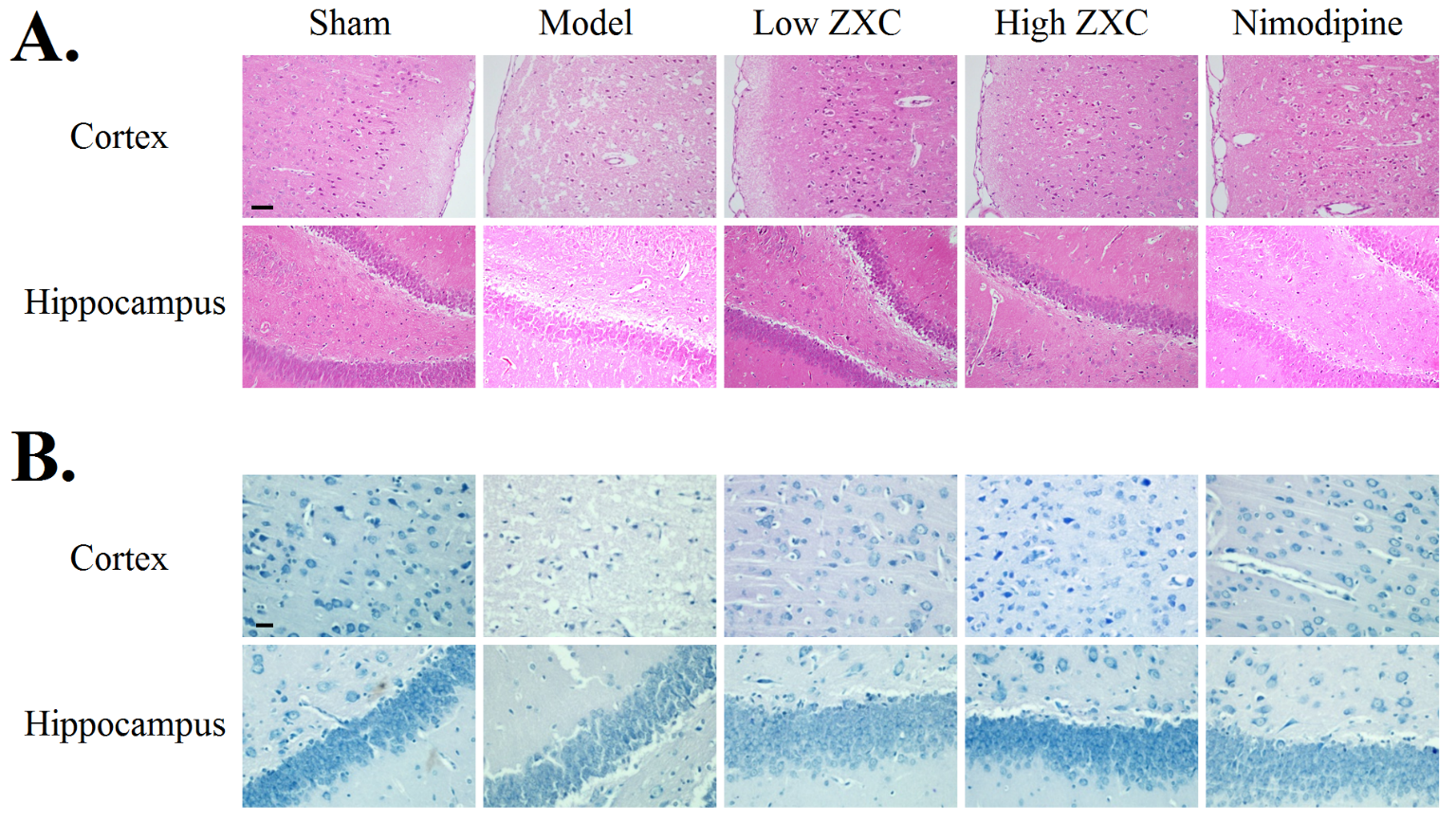


*Figure S2*

Immunohistochemistry of caspase-3 (top row) and p38 (bottom row) for the sham, MCAO model, and ZXC groups at the 90-min ischemia time point (I-90). Scale bar: 50 μm. The magnification of all samples shown here is the same.


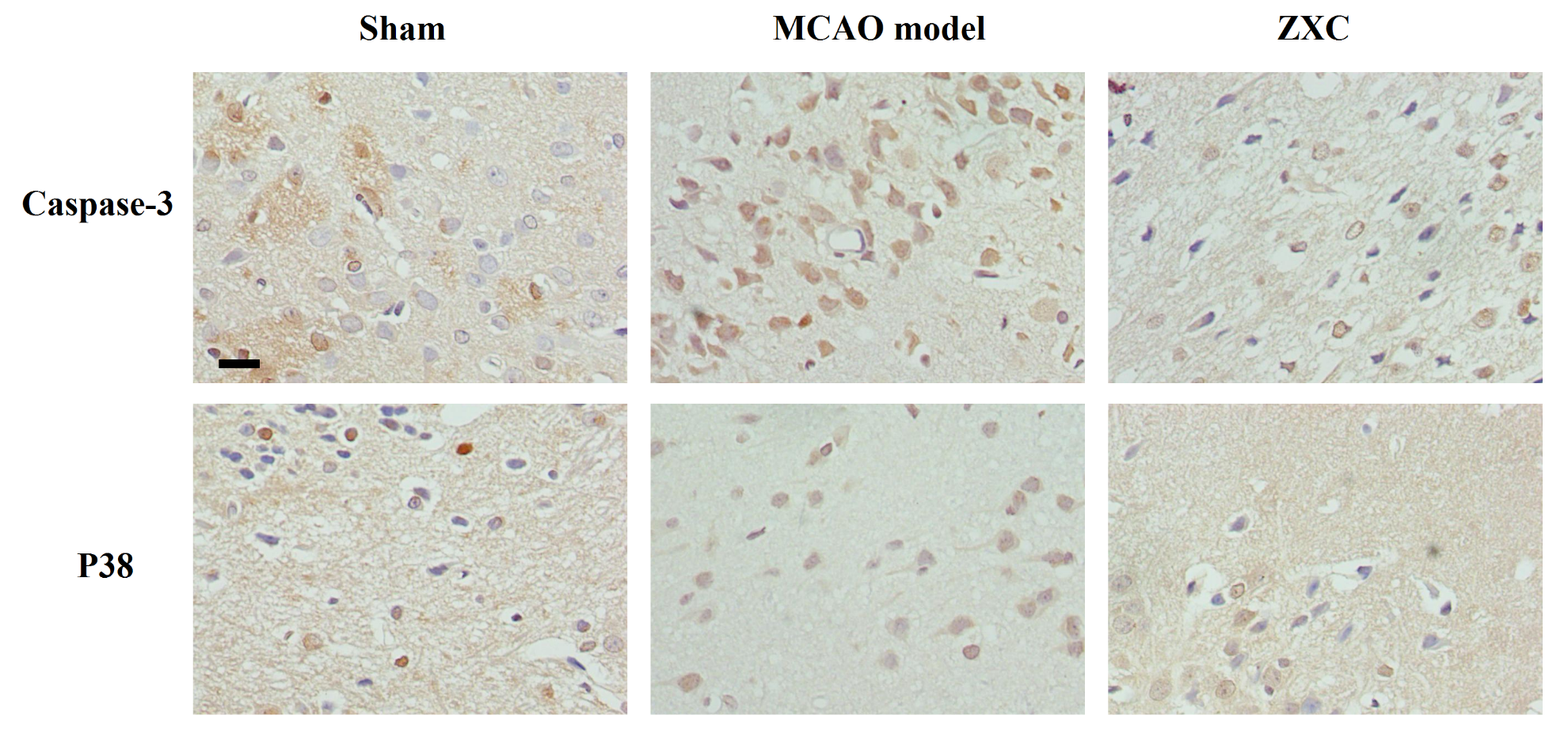

Supplement: Supplementary file 1 [file DataSheet_1.docx]
