# Supplementary material for: Neuroprotective Effects and Mechanisms of Zhenlong Xingnao Capsule in In Vivo and In Vitro Models of Hypoxia
Source: Front Pharmacol. 2019 Sep 26;10:1096. doi: 10.3389/fphar.2019.01096 (PMC6775503; doi:10.3389/fphar.2019.01096)
Supplement: Supplementary file 2 [file DataSheet_2.docx]

**Supplementary tables**

Table S1. Main compounds of ZXC

| Name | Chemical structure |
| --- | --- |
| Gallic acid | 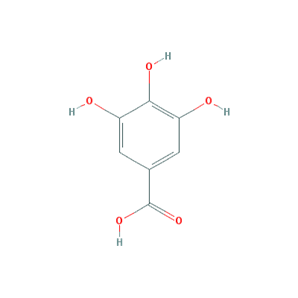 |
| Crocin I | 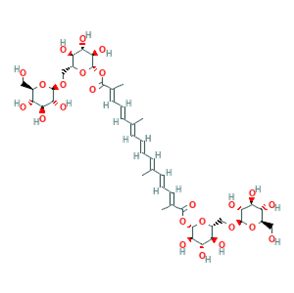 |
| Crocin II | 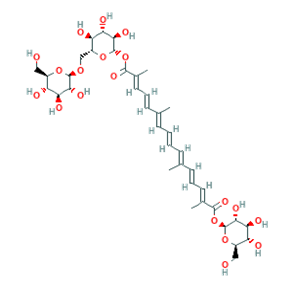 |
| Liquiritin | 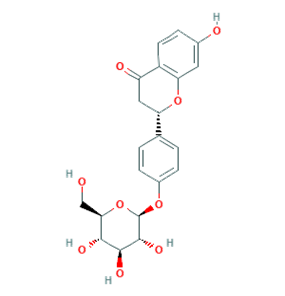 |
| Cinnamic acid | 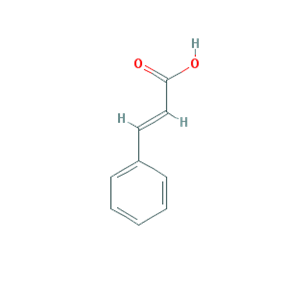 |
| Cinnamaldehyde | 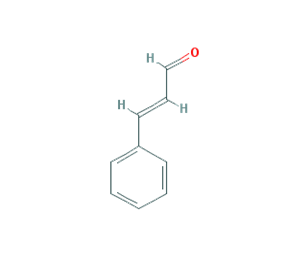 |
| Eugenol | 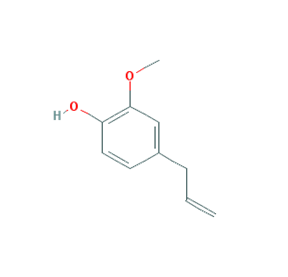 |
| Glycyrrhetinic acid | 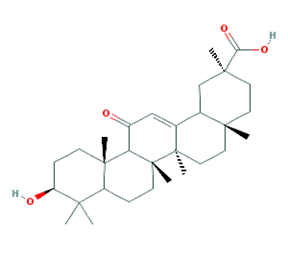 |
| Muscone | 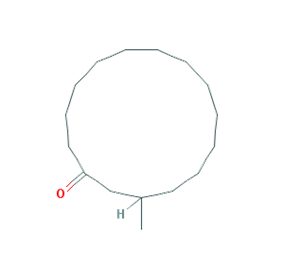 |

ZXC: Zhenlong Xingnao Capsule

Table S2. Real-time polymerase chain reaction primers

| Primer ID | Sequence |
| --- | --- |
| P-P38-F | 5’-AGA CGA ATG GAA GAG CCT GA-3’ |
| P-P38-R | 5’-GGG ATG GAC AGA ACA GAA GC-3’ |
| NF-кB-F | 5’-TGG GAC GAC ACC TCT ACA CA-3’ |
| NF-кB-R | 5’-GGC TCA AAG TTC TCC ACC AG-3’ |
| BCL2-F | 5’-GGT GGA CAA CAT CGC TCT G-3’ |
| BCL2-R | 5’-CAG CCA GGA GAA ATC AAA CA-3’ |
| BAX-F | 5’-ACG CAT CCA CCA AGA AGC-3’ |
| BAX-R | 5’-GCC ACA CGG AAG AAG ACC T-3’ |
| CASPASE3-F | 5’-ACG GGA CTT GGA AAG CAT C-3’ |
| CASPASE3-R | 5’-TAA GGA AGC CTG GAG CAC AG-3’ |
| ACTIN-F | 5’-CCC ATC TAT GAG GGT TAC GC-3’ |
| ACTIN-R | 5’-TTT AAT GTC ACG CAC GAT TTC-3’ |

Table S3. Antioxidant index results for the left-brain tissue (mean ± standard deviation, n = 10)

| Antioxidant index | MDA  (nmol/mg protein) | GSH-Px  (mg/g protein) | T-AOC  (U/mg protein) | T-SOD  (U/mg protein) |
| --- | --- | --- | --- | --- |
| Sham | 2.02±0.86 | 5.68±1.84 | 1.82±0.41 | 4.57±1.73 |
| MCAO model | 2.47±0.94 | 5.98±2.71 | 1.61±0.47 | 3.84±1.43 |
| Low ZXC | 3.23±1.62 | 5.04±1.50 | 1.54±0.56 | 3.98±1.67 |
| High ZXC | 1.35±0.64 | 6.92±2.42 | 1.63±0.55 | 4.02±1.36 |
| Nimodipine | 2.32±0.62 | 5.25±2.06 | 1.65±0.46 | 3.47±1.57 |

MDA: Malondialdehyde; GSH-Px: glutathione peroxidase; T-AOC: total antioxidant capacity; T-SOD: total superoxide dismutase; MCAO: middle cerebral artery occlusion; ZXC: Zhenlong Xingnao Capsule

Table S4. Antioxidant index results for the right-brain tissue (mean ± standard deviation, n = 10)

| Antioxidant index | MDA  (nmol/mg protein) | GSH-Px  (mg/g protein) | T-AOC  (U/mg protein) | T-SOD  (U/mg protein) |
| --- | --- | --- | --- | --- |
| Sham | 1.73±0.62 | 6.31±2.71 | 2.03±0.65 | 5.17±1.83 |
| MCAO model | 2.60±0.82^*^ | 3.83±1.30^**^ | 1.33±0.34^*^ | 3.78±0.88^*^ |
| Low ZXC | 2.23±1.67 | 3.42±1.16 | 1.34±0.40 | 5.50±2.13 |
| High ZXC | 2.23±0.73 | 4.89±1.94 | 1.84±0.50^#^ | 5.58±1.71^#^ |
| Nimodipine | 2.33±1.36 | 4.58±2.18 | 1.38±0.53 | 4.53±1.48 |

^*^: *P* < 0.05 vs. sham group; ^**^: *P* < 0.01 vs. sham group; ^#^: *P* < 0.05 vs. model group.

MDA: Malondialdehyde; GSH-Px: glutathione peroxidase; T-AOC: total antioxidant capacity; T-SOD: total superoxide dismutase; MCAO: middle cerebral artery occlusion; ZXC: Zhenlong Xingnao Capsule
